# Aesthetics over health: stakeholder perceptions of residential window design, indoor ventilation, disease risk, and energy use in urban Dar es Salaam, Tanzania: a mixed-methods study

**DOI:** 10.3389/fpubh.2026.1867864

**Published:** 2026-07-29

**Authors:** Zakia K. Mgaya, Jesca James Mawala, Felista Simon Tarimo, Ibrahim Msuya, Yeromin P. Mlacha, Emmanuel Abraham Mpolya, Dickson Wilson Lwetoijera

**Affiliations:** 1 Training and Capacity Building Department, Ifakara Health Institute, Bagamoyo, Tanzania; 2 School of Life Sciences and Bioengineering (LiSBE), The Nelson Mandela African Institution of Science and Technology (NM-AIST), Arusha, Tanzania; 3 Department of Environmental Health and Ecological Sciences (EHES), Ifakara Health Institute, Ifakara, Tanzania; 4 Department of Health Systems, Impact Evaluation and Policy, Ifakara Health Institute, Dar es Salaam, Tanzania

**Keywords:** aesthetics, built environment, energy use, indoor disease risk, residential ventilation, stakeholder perceptions, urban housing, window design

## Abstract

**Background:**

Residential window design is a primary determinant of natural ventilation in urban housing; however, the factors driving window design choices in rapidly urbanizing sub-Saharan African cities remain poorly understood. In Dar es Salaam Tanzania, sliding aluminum windows have become increasingly dominant in the residential housing market, yet evidence on how stakeholder perceptions of these designs relate to indoor ventilation adequacy, disease risk, and household energy use remains scarce.

**Method:**

This mixed-methods study was conducted in Dar es Salaam Tanzania, between July and October 2025. A structured questionnaire was administered to 611 participants (496 household respondents and 115 hardware store vendors), followed by two focus group discussions with housing stakeholders. Descriptive statistics (frequencies and proportions) summarized participant and household characteristics. Associations between window designs and self-reported diseases and use of artificial cooling systems were examined using chi-square tests and logistic regression, with statistical significance set at a *p*-value < 0.05. Focus group data were analyzed using thematic analysis, and a desktop review was done to assess the presence of multisectoral ventilation guidance.

**Results:**

Sliding aluminum windows dominated the market (57% of sales) and household installations (47%), driven primarily by aesthetic considerations (46%). Awareness of ventilation guidelines was low across all stakeholder groups (18%). Limited airflow was significantly associated with greater reliance on mechanical fans (χ^2^(1) = 5.72, *p* = 0.017). Large household size was the strongest predictor of both malaria (OR = 2.65, 95% CI: 1.61–4.39, *p* < 0.001) and respiratory infections (OR = 3.74, 95% CI: 1.17–12.0, *p* = 0.026). Use of gas as cooking fuel was protective against respiratory infections (OR = 0.35, 95% CI: 0.14–0.85, *p* = 0.021). The desktop review revealed no enforceable benchmarks linking window design to ventilation performance, disease prevention, or energy efficiency in Tanzanian building regulations.

**Conclusion:**

Market dynamics and structural constraints shape window design choices in urban Dar es Salaam more than health-oriented performance standards. Bringing public health ideas into housing rules, creating better ventilation guidelines for specific areas, and raising community awareness could improve indoor air quality and reduce reliance on energy-intensive solutions in rapidly growing cities.

## 1 Introduction

Sustainable building designs are increasingly recognized not only for being eco-friendly but also for their potential as public health interventions (1). Individuals spend approximately 80%−90% of their time indoors, making building environments important determinants of health (2). Architectural features that influence indoor environmental quality including ventilation, natural lighting, and thermal comfort have significant implications for both physical and mental health outcomes (3).

Evidence shows that well-designed windows can improve indoor environmental quality through adequate ventilation and daylight access. Adequate ventilation reduces excess humidity, carbon dioxide, and human odor cues used by mosquitoes during host seeking, thereby reducing malaria transmission risk (4, 5). Natural lighting further contributes to health by inactivating microorganisms such as *Mycobacterium tuberculosis* and SARS-CoV-2, reducing respiratory infection risk (6, 7). Daylight exposure also supports circadian rhythm regulation, mood, and cognitive performance (8). Beyond these health benefits, improved ventilation and daylighting may reduce household energy use by lowering reliance on artificial lighting and mechanical cooling systems (3, 9).

It is important to distinguish between window operability the functional mechanism by which a window opens, such as sliding, casement, hung, or fixed and window design as a broader built-environment concept encompassing window size, placement, material, glazing type, and screening configuration. These two dimensions interact to determine overall ventilation performance. Sliding aluminum windows, for instance, are mechanically limited to opening only half of the available space, integrally restricting airflow regardless of other design features (10). Casement windows, by contrast, can achieve full space opening, allowing greater cross-ventilation. Understanding this distinction is important for interpreting stakeholder perceptions, which may mix operability type with overall design quality. Despite documented performance differences, residential window design choices in many rapidly urbanizing regions are driven by market availability, cost, and aesthetic appeal. Sliding aluminum windows have become increasingly dominant in sub-Saharan African cities owing to their modern appearance and affordability (9), even though they restrict airflow and are frequently installed with tinted glass that limits natural light penetration (11).

While a growing body of research has documented the health implications of window design, little is known about whether these relationships are recognized by those who make housing decisions. Evidence from housing policy research suggests that awareness of health-related housing benefits is an important driver of policy adoption, yet healthy housing standards remain weakly implemented in many low- and middle-income countries. Stakeholder studies show that financial constraints, fragmented governance structures, and limited awareness among decision-makers often hinder the implementation of housing-based health interventions. Understanding stakeholder perceptions of window design is therefore important, as perceptions shape housing choices, construction practices, and policy priorities.

Therefore, addressing these gaps requires approaches that go beyond quantitative measurement to fully capture the lived experiences, decision-making processes, and guideline constraints that shape housing practices. Integrating qualitative and quantitative approaches is therefore important for developing a more complete understanding of stakeholder awareness, behaviors, and the broader context within which housing decisions are made.

This gap is particularly relevant in Dar es Salaam Tanzania, among the fastest growing cities in Africa, with population growth approaching 5% annually (12, 13). Tanzania faces an estimated housing deficit of approximately three million units, increasing by about 200,000 units each year, creating strong pressure for rapid residential construction (14). Much of this growth has occurs in informal settlements where building practices are often guided by material availability and affordability rather than climate-responsive design principles (15, 16). Approximately 70% of residents live in informal housing characterized by limited infrastructure and minimal regulatory oversight (17, 18). The city experiences a tropical coastal climate characterized by persistently high temperatures and relative humidity levels ranging from (70%−85%), making adequate ventilation essential for indoor comfort, air quality, and energy efficiency (19, 20). Tanzania also bears a high burden of both malaria (21) and tuberculosis (22), reinforcing the importance of housing-related environmental factors in shaping infection risk. This study therefore examines stakeholder perceptions of residential window design choices and their implications for indoor ventilation, disease risk, and energy use in urban Dar es Salaam Tanzania.

### 1.1 Specific objectives

1. To describe the patterns and market availability of residential window designs across all five municipalities of Dar es Salaam.
2. To assess stakeholder awareness, knowledge, and perceptions of residential window design and its implications for indoor ventilation and disease risk.
3. To examine associations between window design type and self-reported disease outcomes and household energy use.
4. To review existing multisectoral guidelines on residential window ventilation design in Tanzania.

## 2 Methods

### 2.1 Study area

**Figure 1:**
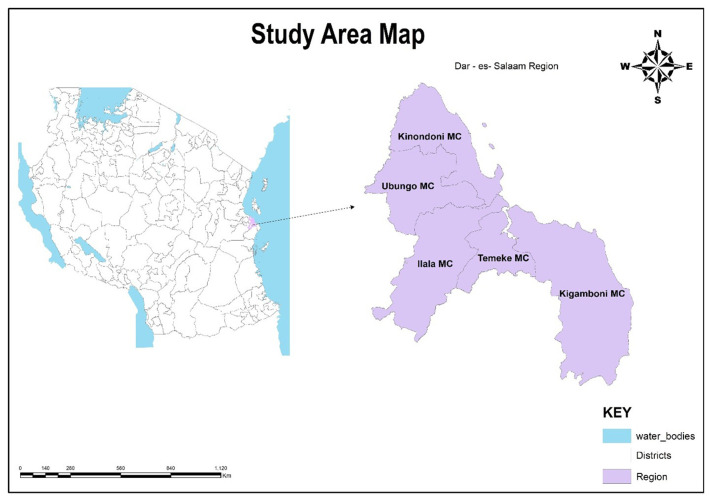
Shows study sites visited during data collection namely: Kinondoni, Temeke, Ubungo, and Kigamboni these are municipals found in Dar es Salaam region.

This study was conducted in Dar es Salaam, the economic capital and largest city in Tanzania, situated at 6°48′S 39°18′E along the Indian Ocean, covering an estimated 1,600 km^2^ across five municipalities: Ilala, Kinondoni, Temeke, Ubungo, and Kigamboni (Figure 1). All five municipalities were included to ensure representative geographic coverage. Dar es Salaam has a population of 8 million people from over 126 ethnic groups, with an estimated 931,707 buildings of which 86% are residential (14, 23). The city experiences a hot and humid tropical climate, with average temperatures ranging between 27 °C and 30 °C and relative humidity between 70 and 80%, making a window design a critical determinant of indoor comfort, energy use, and health.

### 2.2 Study design

This study employed a cross-sectional mixed-methods design, integrating both quantitative and qualitative approaches. The quantitative component consisted of household and market surveys as well as observations. The qualitative components included focus group discussions (FGDs) and a desktop review of the multisectoral guidelines on window ventilation designs. This approach allowed for a comprehensive assessment of awareness, perceptions, and practices related to residential window design, as well as its associated health and energy implications in urban Dar es Salaam Tanzania.

### 2.3 Sampling

The sample size for the quantitative component was determined using Cochran's formula: **N**_**0**_ **=** **Z**^2^^*^p ^*^(1–p) / e^**2**^. Where: Z = 1.96 (for 95% confidence level), *p* = 0.5 (estimated proportion of the population with the characteristic of interest), *e* = 0.05 (margin of error). This yielded a minimum required sample size of 389 households. To improve representativeness and account for potential non-response, the sample was increased to a final total 496 households. A proportionate stratified random sampling technique was applied using the housing census data for Dar es Salaam (NBS,) (23) to distribute the sample across municipalities according to the formula $ni={\frac{Ni}{n}}^{*}n$, with each household being considered as a single sampling unit. For the hardware store vendor component, a purposive sampling approach was used to recruit 115 vendors across all five municipalities, selected based on their direct involvement in the sale and supply of residential window designs.

For the qualitative component, a purposive sampling approach was employed to recruit participants for the FGDs, comprising experts directly engaged in the housing and health sectors. Two FGD sessions were conducted: the first session on 16th October 2025 comprised 9 participants, and the second session on 17th October 2025 comprised 8 participants, giving a total of 17 FGD participants. Stakeholders included homeowners, tenants, architects (civil and electrical), town planners, land and quantity surveyors, and medical doctors, each with a minimum of 2 years of professional experience.

### 2.4 Data collection

The questionnaire and FGD guide were assessed for clarity and relevance to the study objectives. Feedback from the pilot phase was used to revise both tools before formal data collection. Data were collected by trained research investigators through face-to-face interviews using Kobo Collect software on electronic tablets.

The quantitative component comprised three data sources. First, a household survey was conducted between July and August 2025 among adult residents (≥25 years) who had lived in the household for at least 6 months. Structured questionnaires captured sociodemographic characteristics and respondents' awareness, attitudes, and practices related to window design, ventilation, energy use, and indoor disease risk. Second, structured observations were conducted using a checklist attached to the household survey tool to capture housing characteristics, including window type, floor type, roofing material, cooking practices, eave status, and environmental conditions relevant to indoor air quality and mosquito exposure. Observation findings were used to supplement and validate self-reported household data. Third, a market survey was conducted among 115 hardware store vendors to assess the availability, affordability, and market dominance of different window designs, and the extent of advisory support provided to customers.

For the qualitative component, invitations were sent to all participants 1 month before the discussions. FGDs were conducted on 16th and 17th October 2025, moderated by an experienced social scientist fluent in Swahili, with assistance from a note-taker and audio recorder. Discussions explored awareness of building regulations, ventilation-related health risks, energy implications, barriers to adequate ventilation, and recommendations for multisectoral collaboration. Data collection followed a theoretical saturation approach.

### 2.5 Document review

A structured document review was conducted to identify existing multi-sectoral guidelines relevant to residential window-ventilation design in Tanzania. The review searched on national regulatory institutions, including the Ministry of Lands, Tanzania Bureau of Standards, National Construction Council, Architects and Quantity Surveyor Registration Board, Engineers Registration Board, Tanzania Legal Information Institute and Government Gazette. Academic database searches using Google Scholar and PubMed supplemented institutional sources. Search terms included combinations of “Tanzania building regulations,” “ventilation standards,” “window design guidelines,” “natural ventilation,” “residential building code,” and “indoor air circulation requirements,” as well as Boolean combinations such as: “window design” AND “building regulations” AND “Tanzania”; “ventilation” OR “airflow” AND “housing standards”; “construction guidelines” AND “health”; “urban planning” AND “ventilation requirements”; and “material standards” AND “Tanzania Bureau of Standards.” Of 162 records identified, 28 duplicates were removed, 134 underwent title and abstract screening, 38 full texts were assessed for eligibility, and 18 met the inclusion criteria for full analysis using a deductive and inductive content extraction matrix.

### 2.6 Data analysis

#### 2.6.1 Quantitative data analysis

Quantitative data were analyzed using the open-source software STATA version 18. Descriptive statistics (frequencies and proportions) summarized participant and household characteristics. The chi-square test examined the association between window design type and use of mechanical fans for cooling. Two multivariable logistic regression models assessed associations between housing characteristics and self-reported malaria and respiratory infections, respectively, with results expressed as adjusted odds ratios (AOR) with 95% confidence intervals (CI). A separate logistic regression model identified predictors of sliding window installation. Independent variables were selected based on theoretical relevance and prior literature. Statistical significance was set at *p* < 0.05. The total number of respondents varied across questions due to the exclusion of non-respondents and missing data.

#### 2.6.2 Qualitative data analysis

FGD audio recordings were transcribed verbatim and imported into NVivo version 12. Both inductive and deductive coding approaches were applied. Inductive codes were derived from participant transcripts, while deductive codes were developed from the FGD guides and study objectives. Analysis was conducted in Swahili, with representative quotes translated into English. Quantitative and qualitative findings were integrated during the interpretation phase, with quantitative results presented first and qualitative quotes used to contextualize and elaborate on key themes.

## 3 Results

### 3.1 Household and sociodemographic characteristics of the study participants

The study involved 496 household respondents and 115 hardware store vendors across all five municipalities of Dar es Salaam, namely: Kinondoni, Kigamboni, Ilala, Temeke, and Ubungo (Table 1). The household participants were predominantly female (66%), married (73%), and young adults (20–39 years) (61%). Most were self-employed (55%) and held a secondary education (46%). The majority owned their homes (64%), with most households comprising four to five members (41%). All households had iron sheet roofing, cement brick walls (98%), and wooden doors (96%). Most had suspended gypsum ceilings (79%), closed eaves (58%), and concrete flooring (52%). Nearly all households used electricity for lighting (96%) and cooked primarily with gas (81%) (Table 1). The distribution of these characteristics by municipalities is available in Supplementary Table 1.

**Table 1A:** Sociodemographic characteristics of household participants.

Variables	*n* (%)
Gender	
Male	167 (34)
Female	329 (66)
Age group	
Young adults (20–39)	304 (61)
Middle adults (40–59)	159 (32)
Old adults (60–100)	33 (7)
Marital status	
Single	108 (22)
Married	364 (73)
Separated	24 (5)
Education level	
Primary	187 (38)
Secondary	229 (46)
Tertiary	80 (16)
Occupation	
None	145 (29)
Employed	79 (16)
Entrepreneur	271 (55)
Tenure status	
Owned	315 (64)
Rented	181 (36)
Household size	
Small (1–3)	149 (30)
Medium (4–5)	205 (41)
Large ≥ 6	142 (29)
Districts	
Kigamboni	118 (24)
Temeke	118 (24)
Ilala	80 (16)
Kinondoni	100 (20)
Ubungo	80 (16)

**Table 1B:** Household structural and utility characteristics.

Variables	*n* (%)
Roof material	
Iron/Aluminum sheets	496 (100)
Number of layers	
3 layers (grills, mesh/mosquito wire, and glass)	258 (52)
2 layers (combination of two of the three layers)	219 (44)
1 layer (mesh/mosquito wire/glass only)	19 (4)
Window material	
Aluminum	232 (47)
Metal bars	248 (50)
Mesh only	16 (3)
Eaves status	
Completely open	28 (6)
Partially open	180 (36)
Closed	288 (58)
Ceiling	
Gypsum	391 (79)
Wood	18 (3)
None	87 (18)
Door material	
Wood	477 (96)
Iron sheet	13 (3)
Aluminum	6 (1)
Floor material	
Concrete	260 (52)
Wood	1 (0.5)
Tiles	234 (47)
Vinyl	1 (0.5)
Wall material	
Burnt bricks	8 (2)
Cement bricks	488 (98)
Cooking energy	
Charcoal	92 (19)
Gas	404 (81)
Lighting energy	
Electricity	478 (96)
Other (candle, torch, battery)	18 (4)
Cooking place	
Indoors (main house)	383 (77)
Separate kitchen outside	113 (23)

For the qualitative component, a total of 17 participants took part in the FGDs, comprising 11 males and 6 females. Participants were all adults, with a median age of approximately 36 years. The majority held at least a bachelor's degree, with several participants possessing postgraduate qualifications (MSc). Professional experience ranged from 2 to 35 years, reflecting both early-career and senior practitioners. Most participants were unmarried, while a substantial proportion were married.

### 3.2 Hardware store vendor's profile

The vendor sample reflected an experienced workforce: 65% reported more than 3 years of business experience, while 30% had one to 3 years of experience (Table 2). Formal training was nearly absent, with 93% of vendors reporting no professional training. Of the few who received training (7%), most were trained by NGOs (75%). Despite this, two-thirds (63%) of vendors were aware of performance differences between window type, while 20% were unaware and 17% were uncertain.

**Table 2:** Hardware store vendor's profile.

Characteristics of study Vendors	
Variable	*n* (%)
Business experience	
Less than 1 year	5 (4.3%)
1–3 years	35 (30%)
More than 3 years	75 (65%)
Ever trained	
No	107 (93%)
Yes	8 (7%)
The organization that hosted the training	
NGOs	6 (75%)
Technician	1 (13%)
VETA	1 (13%)
Awareness of window performance	
No	23 (20%)
Yes	73 (63%)
Not sure	19 (17%)

### 3.3 Stakeholder perceptions of ventilation and disease risk

Over half of the participants (56%, *n* = 256) recognized that some diseases can spread through the air, while 25% (*n* = 125) understood how window design affects indoor air flow. Nearly 43% were unsure how window design affects ventilation and disease transmission risk, while 58% believed window design does not play a role in preventing airborne diseases (Table 3).

**Table 3:** Knowledge of window design and disease risk among household respondents.

Knowledge statement	Response category	*n* (%)
Are you aware that some diseases can be transmitted due to poor indoor ventilation?	Yes	253 (56)
	No	200 (44)
Do you think the amount of airflow through your window might affect the risk of disease transmission?	Yes, significantly – poor ventilation increases risk	123 (25)
	Not sure	231 (46)
	No – ventilation doesn't matter	143 (29)
How important do you think proper window design is in preventing the risk of disease spread?	Important	184 (41)
	Not important	265 (58)
	Not sure	4 (1)

Similarly, qualitative findings revealed widespread uncertainty about the mechanical link between window design and health vulnerability. Only medically trained FGD participants could clearly explain the health risks of poor ventilation, while most community members demonstrated limited awareness.

“*The community does not really know which housing conditions increase disease risk. People assume they are safe as long as the house appears nice. They do not connect ventilation with disease unless someone falls sick.”* (**TCU leader, female**)

### 3.4 Residential window design patterns and market availability

**Figure 2:**
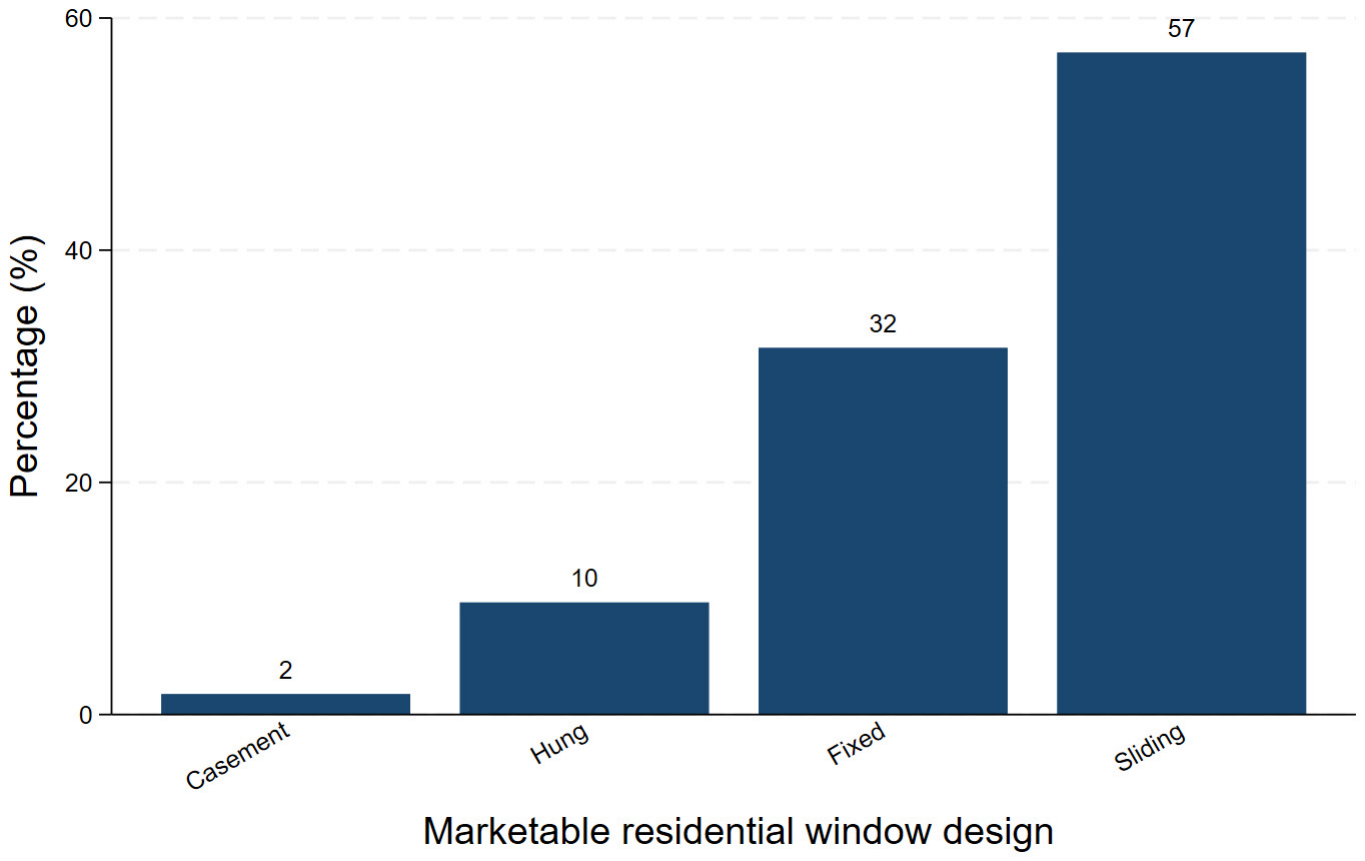
Distribution of residential window designs sold by hardware store vendors in Dar es Salaam Tanzania (*n* = 115). Sliding aluminum windows dominated the market at 57% of all sales, followed by fixed windows at 32%, hung windows at 10%, and casement windows at 2%.

The market survey showed that window supply in Dar es Salaam is heavily concentrated around two designs: sliding aluminum designs (57% of all sales) and fixed windows (32%), together accounting for nearly 90% of the market (Figure 2). Casement windows represented only 2% and hung windows 10% of market sales. This market distribution closely mirrored household installation patterns, where fixed windows were slightly more common (50%, *n* = 248) than sliding aluminum windows (47%, *n* = 232). Most households installed multi-layered window configurations: 52% used a three-layer combination (metal grill + mosquito mesh + glass) and 44% used a two-layer combination (Table 2).

### 3.5 Drivers shaping residential window design choices

**Figure 3:**
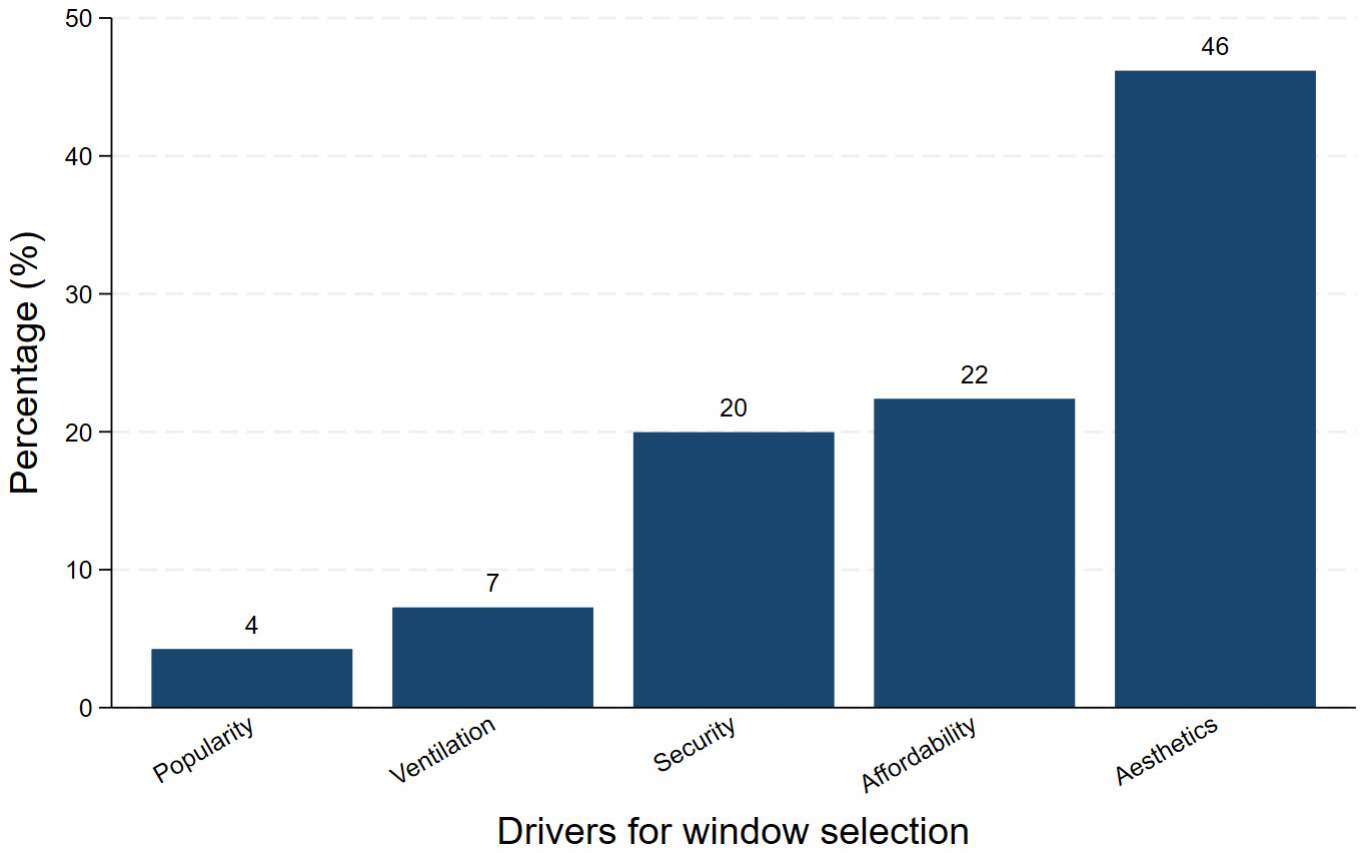
Factors influencing residential window design selection among household respondents in Dar es Salaam Tanzania (*n* = 496). Aesthetics was the dominant driver (46%), with ventilation cited by only 7% of respondents.

Window design selection was primarily driven by aesthetic considerations (46%), followed by affordability (22%), security (20%), and popularity (4%). Ventilation performance was cited by only 7% of respondents as a primary reason for window selection (Figure 3).

Qualitative findings strongly reinforced these patterns. Majority of homeowners described aesthetics and cost as the dominant drivers, often at the expense of ventilation performance. Moreover, in the rental market, agents (dalali) commonly promoted sliding aluminum windows as markers of a “modern” housing.

“*Most people don't choose windows based on airflow or health; many choose sliding aluminum because they seem modern and fashionable.”* (**Homeowner, Male**)

### 3.6 Association between window designs and self-reported disease outcomes

**Figure 4:**
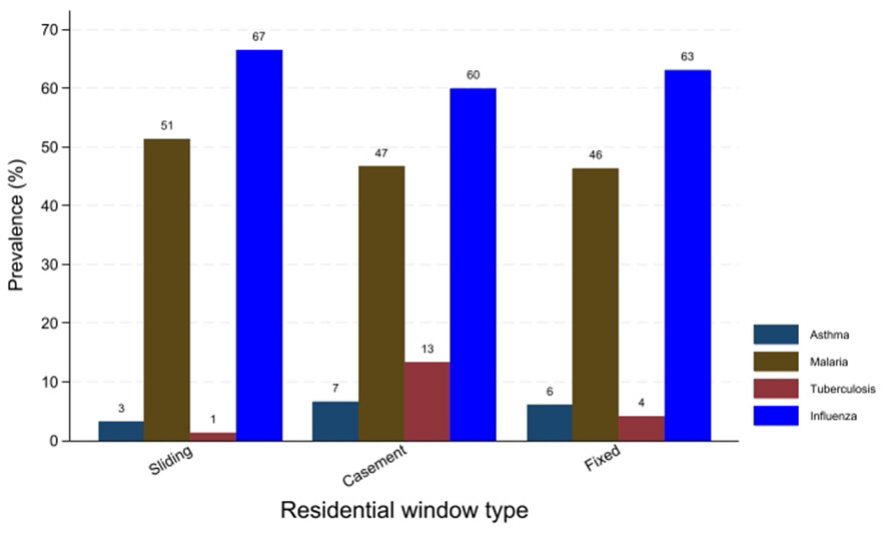
Prevalence of self-reported diseases by residential window type among household respondents in Dar es Salaam Tanzania (*n* = 496). Disease categories include influenza, malaria, tuberculosis, and asthma. Influenza was the most prevalent self-reported condition across all window types.

Multivariable logistic regression was performed to assess housing predictors of self-reported malaria and respiratory infections (Table 4). Window design type was not a statistically significant independent predictor of either disease outcome, though sliding windows showed a borderline association with malaria (AOR = 1.65, 95% CI: 0.97–2.81, *p* = 0.064). Large household size was the strongest and most consistent predictor across both disease outcomes: residents of large households had 2 times higher odds of malaria (AOR = 2.65, 95% CI: 1.61–4.39, *p* < 0.001) and 3 times higher odds of respiratory infections (AOR = 3.74, 95% CI: 1.17–12.0, *p* = 0.026) compared to those in small households. Households using gas as their primary cooking fuel had significantly lower odds of respiratory infections compared to those using solid fuels (AOR = 0.35, 95% CI: 0.14–0.85, *p* = 0.021). Secondary education showed a suggestive but non-significant protective association with respiratory infections (AOR = 0.45, 95% CI: 0.19–1.10, *p* = 0.081). All remaining variables showed no statistically significant association with either outcome (all *p* > 0.05). The prevalence of self-reported diseases by window type is illustrated in Figure 4.

**Table 4A:** Logistic regression for factors associated with malaria.

Malaria	Odds ratio	95% conf. interval	*p*-value
Window type			
Sliding	1.65	0.97–2.81	0.064
Education			
Secondary	0.72	0.47–1.11	0.14
Tertiary	0.83	0.46–1.51	0.538
Occupation			
Employed	1.21	0.65–2.23	0.547
Entrepreneur	1.23	0.79–1.91	0.353
Floor			
Tiles	0.8	0.51–1.25	0.329
Window layers			
Mesh wire	0.84	0.35–2	0.694
Iron bars	1.18	0.66–2.1	0.579
Mosquito mesh	1.21	0.65–2.24	0.554
Ceiling type			
Gypsum	1.11	0.61–2	0.733
Wood	1.63	0.53–5.06	0.396
Eaves status			
Partially open	1.07	0.41–2.78	0.888
Completely closed	0.9775	0.41–2.36	0.96
Cooking fuel			
Gas	0.9	0.53–1.55	0.712
Household size			
Medium	1.42	0.91–2.23	0.127
Large	2.65	1.61–4.39	0
Breeding site			
Yes	0.96	0.57–1.63	0.882
Districts			
Kigamboni	0.96	0.62–1.5	0.872
_Cons	0.51	0.15–1.7	0.273

**Table 4B:** Logistic regression for factors associated with respiratory illness outcomes.

Variable	Odds ratio	95% confidence interval	*P*-value
Window type			
Sliding	0.537	0.20–1.43	0.214
Education level			
Secondary	0.452	0.19–1.10	0.081
Tertiary	0.859	0.26–2.82	0.802
Districts			
Kigamboni	1.272	0.42–3.85	0.67
Ilala	0.297	0.07–1.29	0.106
Kinondoni	1.361	0.46–4.06	0.58
Ubungo	1.744	0.40–7.56	0.458
Occupation status			
Employed	0.746	0.18–3.16	0.69
Entrepreneur	0.988	0.43–2.26	0.978
Floor type			
Tiles	2.225	0.875–5.66	0.093
Ceiling status			
Gypsum	0.552	0.20–1.55	0.26
Wood	0.447	0.05–4.38	0.489
Wall type			
Plastered	0.262	0.03–2.69	0.26
Cooking fuel			
Gas	0.35	0.14–0.85	0.021
Household size			
Medium	2.114	0.69–6.47	0.19
Large	3.742	1.17–12.00	0.026
Constant (_cons)	0.507	0.04–6.70	0.606

### 3.7 Association between window designs and use of the artificial ventilation system

Of the surveyed households, none reported using air conditioners for ventilation support; instead, fans were the primary alternative ventilation system. A significant association (χ^2^(1) = 5.72, *p* = 0.017) was observed between window design and fan use. Households with sliding aluminum windows were more likely to use fans for cooling (55%) than households with fixed windows (45%) (Table 5).

**Table 5:** Association between residential window design type and the use of a fan for cooling.

Usage of a fan for cooling	Yes	No	Total
	*n* (%)	*n* (%)	*N*
Window design			
Sliding aluminum window	114 (55)	118 (44)	232
Non-sliding window	95 (45)	153 (56)	248
TOTAL	209 (100)	271 (100)	480

In the same vein, the majority of participants consistently described sliding aluminum windows as poor performers in indoor airflow;

“*If we use a sliding aluminum window design here in Dar es Salaam, we must accept that we will also need either a fan or an AC inside the house. Those sliding aluminum windows allow only a small amount of air to enter, so for someone to live comfortably, they must install fans and other mechanical systems*.” (**Electrical engineer, Male**)

A few participants also shared their experience living in houses with sliding aluminum windows, describing them as airless and having inadequate airflow.

“*I have experienced this personally when visiting someone's house; those sliding aluminum windows make you feel as if you are inside a vacuum. The air feels heavy and still, and only when you step outside do you feel relief. After that experience, I realized that with my lifestyle, I would never be able to tolerate living in a house with that kind of window design.”* (**Homeowner, Female**)

### 3.8 Predictors of residential window design installation

Multivariable logistic regression identified several independent predictors of sliding window installation (LR χ^2^(15) = 271.33, *p* < 0.001; pseudo-*R*^2^ = 0.41; AUC = 0.89) (Table 5). Respondents with secondary education had significantly higher odds of installing sliding windows compared to those with primary education (AOR = 1.88, 95% CI: 1.07–3.31, *p* = 0.029). Employed respondents were more likely to install sliding windows than unemployed respondents (AOR = 1.84, 95% CI: 1.24–3.70, *p* = 0.006). Households with tiled floors had higher odds of sliding window installation (AOR = 2.14, 95% CI: 1.24–3.70, *p* = 0.006). The presence of a gypsum ceiling was the strongest predictor (AOR = 32.84, 95% CI: 7.22–149.14, *p* < 0.001), reflecting that sliding aluminum windows are part of a broader aesthetic modernization package. Among reasons for window selection, aesthetics (AOR = 12.70), security (AOR = 5.72), and popularity (AOR = 3.96) were each significantly associated with sliding window installation compared to affordability. Ventilation was not a significant predictor (AOR = 0.71, *p* = 0.710). Geographically, respondents from Ubungo (AOR = 5.33) and Kigamboni (AOR = 2.42) had significantly higher odds of sliding window installation than those from Temeke (Table 6).

**Table 6:** Multivariable logistic regression: predictors of sliding window installation among respondents.

Window type	Adjusted odds ratio	95% confidence interval	*p*-value
Education			
Primary	1	–	–
Secondary	1.88	1.07–3.31	0.029
Tertiary	1.58	0.74–3.40	0.236
Districts			
Temeke	1	–	–
Kigamboni	2.42	1.18–4.94	0.015
Ilala	1.16	0.47–2.83	0.739
Kinondoni	0.90	0.41–1.96	0.793
Ubungo	5.33	2.07–13.72	0.001
Occupation			
None	1	–	–
Employed	1.84	1.24–3.70	0.006
Entrepreneur	0.86	0.48–1.53	0.617
Floor			
Cement	1	–	–
Tiles	2.14	1.24–3.70	0.006
Ceiling			
None	1	–	–
Gypsum	32.84	7.22–149.14	0.00
Wood	3.65	0.39–33.82	0.254
Choices of the window			
Affordability	1	–	–
Aesthetics	12.70	5.89–27.36	0.00
Security	5.72	2.29–14.23	0.00
Ventilation	0.71	0.12–4.05	0.71
Popularity	3.96	1.06–14.72	0.039
_Cons	0.002	0.00–0.01	0.00

### 3.9 Awareness and use of construction guidelines

Awareness of formal construction guidelines related to window design and ventilation was uniformly low across all stakeholder groups. Only 10% of surveyed households and 18% of hardware vendors reported familiarity with any building regulation, and among those who did, knowledge was largely limited to permit requirements rather than technical ventilation standards (Table 7). Construction professionals acknowledged the existence of regulatory documents but indicated limited practical guidance for residential window design.

**Table 7:** Proportion of participants reporting awareness of any building guidelines related to window design and ventilation.

Participant category	Aware	Unaware
	*n* (%)	*n* (%)
Homeowners	50 (10)	446 (90)
Hardware store vendors	21 (18)	94 (82)

“*Everything is entering the market without guidance. People are just buying what they see is popular, not because it suits the climate. And you find that the guidelines only exist somewhere in offices, but ordinary people or private experts can't practice them. Unless someone works with the council or a reputable firm, nonetheless, the rest build based on guesswork.”* **(Architect, Male)**

### 3.10 Construction practices shaping residential window design choices

Survey findings showed that window design decisions were mostly made without professional input. A total of 84% of respondents (*n* = 379) selected their window designs independently, and 93% (*n* = 422) had never received guidance on the importance of ventilation for health or energy efficiency (Table 8). 90 percent had made no airflow-related modifications after moving in, and 96% had not participated in any housing-health community programme in the previous year.

**Table 8:** Practices of study participants regarding window designs decisions.

Practices statement	Yes, *n* (%)	No, *n* (%)
Did you seek professional advice regarding your current window type?	74 (16)	379 (84)
Have you ever received guidance on the importance of ventilation?	31 (7)	422 (93%)
Have you made any changes to your window designs to improve the indoor ventilation?	50 (10)	446 (90)
Have you ever participated in any community programs about healthy housing within the past 12 months?	18 (4)	478 (96)

Similarly, qualitative findings identified four interrelated factors influencing poor ventilation practices during construction: limited access to professional guidance, attributed by cost-related avoidance of professional construction experts, which in turn increases reliance on unskilled local construction experts. Together, these factors resulted in a window design decision that prioritized affordability and trends over ventilation performance.

“*Most people don't ask professionals. They think calling experts is expensive, so they only ask an architect to draw something and stamp it, and the rest of the building work is given to local experts, thus ventilation is never explained to them.”* (**Architect, Male**)

### 3.11 Attitude toward indoor air circulation and health

Majority (83%) of survey respondents believed poor ventilation contributes to disease spread, and 82% perceived a health risk to their family from poor indoor air circulation (Table 9). Strong policy support was evident: 90% approved of the enforcement of housing ventilation standards and 87% expressed interest in learning about home ventilation improvements. However, only 38% were likely to seek professional advice, with 53% remaining unsure. This gap between positive attitudes and low action readiness reflects the systemic and economic constraints identified throughout this study.

**Table 9:** Attitude toward indoor air circulation and health among households' respondents.

Attitude statements	Yes, *n* (%)	Not-sure, *n* (%)	No, *n* (%)
Do you think poor ventilation inside homes can contribute to the spread of diseases?	410 (83)	66 (13)	20 (4)
Do you believe your health or your family's health could be at risk due to poor indoor air circulation	371 (82)	54 (12)	28 (6)
Would you be willing to make changes to your home's window/ventilation system	344 (76)	32 (7)	77 (17)
Will you support the effective enforcement of housing ventilation standards?	406 (90)	35 (7)	12 (3)
How likely are you to seek professional advice on ventilation guidance?	172 (38)	239 (53)	42 (9)
In your opinion, do people in your community understand the health risks due to poor indoor air circulation?	213 (47)	110 (24)	130 (29)
Would you be interested in learning how to improve ventilation in your home?	434 (87)	0 (0)	62 (13)

Similarly, FGDs further revealed overall positive attitudes toward ventilation and its role in health. The majority of participants consistently acknowledged that poor airflow contributes to discomfort and increases the risk of disease spread. However, a few participants also highlighted a persistent gap between awareness and practice.

“*Ventilation is important, but most of us only think about it when something goes wrong, like when the house becomes too hot, or someone gets respiratory issues. Before that, our priorities are more on cost or than proper airflow.”* (**Homeowner, Male**)

### 3.12 Landscape of existing multi-sectoral guidelines on residential window design

The document review identified national and municipal regulatory documents governing residential construction in Tanzania; however, explicit guidance on residential window design and ventilation performance was severely limited. Of 162 records identified, 18 met eligibility criteria for full analysis. Existing building regulations primarily emphasized structural safety, plot coverage, and general habitability, with minimal specification on window size, operability, placement, or airflow requirements relevant to tropical climates. No document provided enforceable benchmarks linking window design to ventilation efficiency, disease prevention, or household energy use.

“*From my perspective, I know the regulations exist, but if we look at people, most of them have no access to this information. Someone builds without knowing the procedures or standards, and later they face problems that could have been avoided if they had received proper guidance.”* **(Homeowner, Female)**

### 3.13 Perceived barriers to achieving adequate indoor ventilation

Participants identified both structural and economic barriers to achieving adequate indoor ventilation. Structural barriers included rapid population growth, rising land values, dense unplanned settlements, and weak enforcement of building regulations. Economic barriers included cost-related avoidance of professional construction expertise and dependence on unskilled local builders who prioritize affordability over ventilation performance.

“*The urban population growth increases tremendously, but the urban planning infrastructure has not improved. Land has become very expensive. People build wherever they can find space. Once a plot of land is acquired, the owner typically aims to maximize the built area to obtain the highest number of rooms without leaving adequate open space. The result is crowded buildings with small windows and no air circulation, since there is no place to put a bigger window***.” (Land surveyor, Male)**

### 3.14 Recommendations on sustaining a multisectoral collaboration

Participants emphasized the need for meaningful multisectoral collaboration across areas such as urban planning, public health, construction, and local government. Key recommendations centered on establishing localized, climate-appropriate standards, combined with stronger enforcement and clear professional accountability, as well as the formal inclusion of public health experts in building permit review teams.

“*The planning team should include someone from the health sector. They can advise when something is not safe, like building too close to a wall or keeping animals near the window.”* (**Land surveyor, Male**)

Furthermore, participants advocated for routine public education and community training on the importance of ventilation for indoor disease prevention and household energy costs reduction.

“*People should be taught early so they know the importance of ventilation. Many mistakes happen simply because we were never given proper guidance.”* **(Homeowner, Female)**

## 4 Discussion

This mixed-methods study provides baseline evidence on residential window design patterns, ventilation practices, and health-related perceptions among housing stakeholders in Dar es Salaam Tanzania. The findings reveal a consistent pattern across quantitative and qualitative findings: aesthetic considerations and market availability drove window design choices more than health or ventilation performance a finding with important implications for indoor disease risk and household energy burden. This core finding aesthetics over health operates across individual households, the hardware market, construction professionals, and the regulatory landscape, constituting a systemic rather than individual-level problem.

Furthermore, the dominance of sliding aluminum and fixed window designs in both market sales and household installations, accounting for nearly 90%: reflects a broader pattern documented in other rapidly urbanizing tropical cities. Similar shifts away from traditional louver and wooden casement window designs toward sliding aluminum windows have been documented in cities such as Accra in Ghana (9) and hot-humid regions across Southeast Asia (10). This cross-regional convergence suggests that the adoption of sliding aluminum windows is driven more by globalized construction trends and evolving housing aspirations than by local climatic responsiveness. Windows are increasingly functioning as markers of economic status and modernity rather than as passive ventilation systems for managing indoor climate (24, 25). Recent studies further suggests that when window choices are based on appearance, residents are less likely to use them in ways that support natural ventilation (26).

The association between sliding window installation and reliance on mechanical fans provides objective proxy evidence that the functional limitations of these windows translate directly into greater energy dependence. Previous studies explained that constrained window openings reduce airflow (10, 27, 28) and that households with sliding aluminum windows tend to incur higher energy costs than those using wooden louvers or casement windows (9). The qualitative data strongly contextualize this finding: residents consistently described sliding aluminum window environments as airless and prone to heat accumulation, requiring fans or air conditioning for basic thermal comfort. This energy costs rises disproportionately on lower-income households who are also least able to afford alternatives escalating inequality with significant implications for energy poverty in rapidly urbanizing sub-Saharan African cities.

Moreover, regression analysis further revealed that household size was a consistent determinant of both malaria and respiratory infections. This is biologically plausible, as crowding intensifies mosquito host-seeking cues while simultaneously facilitating airborne pathogen transmission and reduce adherence to bednet usage (4, 22, 29). The protective effect of gas cooking fuel against respiratory infections reinforces established evidence linking solid fuel combustion to indoor air pollution and respiratory morbidity (6, 29). Although sliding window design showed a borderline association with malaria, this did not reach statistical significance, likely reflecting the study's cross-sectional designs and complex multifactorial nature of disease transmission. The absence of significant associations between window screening, eaves condition, and disease outcomes suggests that sociodemographic and behavioral factors may be more proximate determinants of self-reported disease than structural housing features alone. These findings do not diminish the public health relevance of window design but highlight that its effects operate within a broader web of social, behavioral, and environmental determinants.

Insufficient awareness of local building guidelines was evident among homeowners, tenants, and hardware store vendors, consistent with evidence from other low- and middle-income settings, that climate-responsive residential guidelines suffer from uneven enforcement and limited dissemination (12, 18). The document review finding that no enforceable benchmarks linking window design to ventilation performance, disease prevention, or energy use exist in Tanzanian building regulations is among the most policy-significant contributions of this study. This regulatory void means that even stakeholders who wish to make health-oriented window choices have no prescriptive framework to guide or incentivize them. Construction professionals appeared to rely on imported standards that poorly reflect local environmental and urban realities, while hardware vendors the most accessible point of consumer guidance operated with almost no formal training.

Despite recognition that poor ventilation poses health risks and strong interest in learning about ventilation improvements, few respondents were likely to seek professional advice. This knowledge-action gap reflects systemic and economic constraints rather than a lack of awareness: financial limitations, insecure tenure, limited access to professional guidance, and the absence of accessible regulatory support restrict residents' ability to translate health awareness into design change (2, 30, 31). Structural barriers to adequate indoor ventilation including dense unplanned settlement patterns, rapid population growth, overcrowding, and weak regulatory enforcement further limit what individual households can achieve through window design choices alone (20, 32, 33). These findings underscore that inadequate ventilation in urban Dar es Salaam is a systemic outcome shaped by urban growth patterns and governance shortcomings, not merely a product of individual household decisions.

### 4.1 Strength

This study addresses the underexplored intersection of the built environment and public health by examining residential window design choices in an urban sub-Saharan African context through a mixed-methods lens. The use of multiple stakeholder groups households, hardware vendors, and housing professionals provides a system-wide perspective on how window design decisions are influenced across the housing value chain. The integration of a regulatory document review with primary data collection is a methodological strength that few comparable studies have employed, providing a unique perspective on the governance dimension of indoor ventilation. Lastly, conducting the study in a rapidly growing tropical city with a relatively high burden of vector-borne and respiratory infectious diseases strengthens the contextual and public health relevance of the findings.

### 4.2 Study limitations

This study has several limitations that should be considered when interpreting the findings. First, data on ventilation awareness, window use, and housing decisions were primarily derived from self-reported surveys and FGDs, which may not fully capture actual airflow performance or occupant behaviors. Unlike studies that employed objective airflow measurements such as anemometry or tracer gas techniques to quantify ventilation performance (10, 27), this study relied on perceived ventilation adequacy and proxy indicators such as fan use. Future studies should incorporate objective airflow measurements to complement perception-based evidence.

Second, the cross-sectional design limits causal inference between window design characteristics and health outcomes, consistent with limitations acknowledged in similar housing and health studies (30, 34). Third, disease data were based on self-report rather than clinically confirmed diagnoses, which may introduce recall or social desirability bias. Fourth, the borderline statistical association between sliding windows and malaria suggests the study may have been insufficiently powered to detect a significant association for this specific outcome, and adequately powered longitudinal studies are necessary. Lastly, the study was confined to Dar es Salaam, constraining generalizability to other Tanzanian cities with different climatic, regulatory, or socioeconomic conditions. Nevertheless, the consistency of results with regional and international literature supports their broader relevance for rapidly urbanizing tropical contexts.

## 5 Conclusion

This study demonstrates that residential window design choices in urban Dar es Salaam are driven primarily by aesthetics and market availability rather than health-oriented ventilation performance. This aesthetic-over-health dynamic contributes to limited natural ventilation, increased artificial ventilation systems dependence, indoor thermal discomfort, and potential disease transmission risk, with particular implications for household energy burden and public health in a rapidly urbanizing tropical city. Although residents broadly recognize that poor ventilation has adverse health consequences, this awareness rarely translates into informed housing or retrofit decisions due to structural, economic, and governance constraints. These findings underscore the urgent need for context-specific, enforceable building guidelines that integrate natural ventilation performance, energy efficiency standards, and disease-reducing measures into tropical urban housing policy. Adapting locally used window designs through improved operability, size, placement, and screening alongside community sensitization and meaningful multisectoral collaboration between public health, urban planning, construction, and regulatory sectors offers a realistic pathway to healthier, more energy-efficient housing in rapidly urbanizing sub-Saharan African cities.

### 5.1 Recommendations

#### 5.1.1 Policy and regulatory implications

The document review finding that no enforceable ventilation benchmarks exist in Tanzanian building regulations (Section 3.12) underscores the urgency of developing locally tailored, climate-responsive residential building guidelines that explicitly address natural ventilation requirements, window operability standards, and dense urban form. Policymakers should prioritize the development, dissemination, and enforcement of practical ventilation-oriented standards suitable for tropical climates, with particular attention to ensuring these reach informal housing developers and self-builders who constitute the majority of Dar es Salaam's residential construction sector. Integrating public health evidence into building regulations may align housing design with disease prevention and energy efficiency goals.

#### 5.1.2 Housing practice and market engagement

Given that 93% of hardware vendors received no formal training and that aesthetics drove 46% of window selection decisions (Section 3.5), hardware store vendors represent the most accessible and impactful intervention point for promoting health-oriented window design choices. Targeted training programs for vendors combined with simple, locally adapted guidance materials on window installation and ventilation principles could meaningfully shift market-level decision-making toward health-oriented design without requiring substantial household expenditure.

#### 5.1.3 Future research

Further research is needed to quantify airflow performance and indoor microclimate conditions associated with common window designs in dense tropical settings. Longitudinal and intervention-based studies could also explore how improvements in window design influence health outcomes, energy use, and window ventilation choices over time. Research examining the cost-effectiveness of simple design modifications, such as adding mesh screening or increasing window aperture size, would provide actionable evidence for both policymakers and housing practitioners in rapidly urbanizing African cities.

## Data Availability

The raw data supporting the conclusions of this article will be made available by the authors, without undue reservation.
