# Supplementary material for: Aesthetics over health: stakeholder perceptions of residential window design, indoor ventilation, disease risk, and energy use in urban Dar es Salaam, Tanzania: a mixed-methods study
Source: Front Public Health. 2026 Jul 29;14:1867864. doi: 10.3389/fpubh.2026.1867864 (PMC13464416; doi:10.3389/fpubh.2026.1867864)
Supplement: Supplementary file 1 [file Table_1.DOCX]

| **Table** 1: Sociodemographic characteristics of study participants by districts | | | | | | |
| --- | --- | --- | --- | --- | --- | --- |
|  | Districts | | | | | |
|  | **Kigamboni** | **Temeke** | **Ilala** | **Kinondoni** | **Ubungo** | **Total** |
| N | 118 (24%) | 118 (24%) | 80 (16%) | 100 (20%) | 80 (16%) | 496 (100%) |
| **Gender** |  |  |  |  |  |  |
| Male | 33 (28%) | 42 (36%) | 29 (36%) | 35 (35%) | 28 (35%) | 167 (34%) |
| Female | 85 (72%) | 76 (64%) | 51 (64%) | 65 (65%) | 52 (65%) | 329 (66%) |
| **Age** **group** |  |  |  |  |  |  |
| Young adults | 84 (71%) | 71 (60%) | 40 (50%) | 51 (51%) | 58 (73%) | 304 (61%) |
| Middle_adults | 29 (25%) | 42 (36%) | 35 (44%) | 36 (36%) | 17 (21%) | 159 (32%) |
| Old_adults | 5 (4.2%) | 5 (4.2%) | 5 (6.3%) | 13 (13%) | 5 (6.3%) | 33 (6.7%) |
| **Marital** **status** |  |  |  |  |  |  |
| Single | 27 (23%) | 30 (25%) | 8 (10%) | 24 (24%) | 19 (24%) | 108 (22%) |
| Married | 84 (71%) | 84 (71%) | 71 (89%) | 67 (67%) | 58 (73%) | 364 (73%) |
| Separated | 7 (5.9%) | 4 (3.4%) | 1 (1.3%) | 9 (9%) | 3 (3.8%) | 24 (4.8%) |
| **Education** **level** |  |  |  |  |  |  |
| Primary | 33 (28%) | 58 (49%) | 38 (48%) | 48 (48%) | 10 (13%) | 187 (38%) |
| Secondary | 62 (53%) | 49 (42%) | 35 (44%) | 34 (34%) | 49 (61%) | 229 (46%) |
| Tertiary | 23 (19%) | 11 (9.3%) | 7 (8.8%) | 18 (18%) | 21 (26%) | 80 (16%) |
| **Occupation** |  |  |  |  |  |  |
| None | 36 (31%) | 40 (34%) | 21 (26%) | 26 (26%) | 22 (28%) | 145 (29%) |
| Employed | 24 (20%) | 19 (16%) | 12 (15%) | 11 (11%) | 13 (16%) | 79 (16%) |
| Entrepreneur | 58 (49%) | 59 (50%) | 47 (59%) | 63 (63%) | 44 (56%) | 271 (55%) |
| **Tenure** **status** |  |  |  |  |  |  |
| Owned | 69 (58%) | 62 (53%) | 65 (81%) | 65 (65%) | 54 (68%) | 315 (64%) |
| Rented | 49 (42%) | 56 (47%) | 15 (19%) | 35 (35%) | 26 (33%) | 181 (36%) |
| **Household size** |  |  |  |  |  |  |
| Small | 39 (33%) | 36 (31%) | 13 (16%) | 24 (24%) | 37 (46%) | 149 (30%) |
| Medium | 45 (38%) | 58 (49%) | 33 (41%) | 30 (30%) | 39 (49%) | 205 (41%) |
| Large | 34 (29%) | 24 (20%) | 34 (43%) | 46 (46%) | 4 (5%) | 142 (29%) |
